# Water quality assessment in mosquito breeding habitats based on dissolved organic matter and chlorophyll measurements by laser-induced fluorescence spectroscopy

**DOI:** 10.1371/journal.pone.0252248

**Published:** 2022-07-27

**Authors:** Andrew A. Huzortey, Andreas A. Kudom, Ben A. Mensah, Baah Sefa-Ntiri, Benjamin Anderson, Angela Akyea

**Affiliations:** 1 Laser and Fibre Optics Centre, Department of Physics, University of Cape Coast, Cape Coast, Ghana; 2 Department of Conservation Biology and Entomology, University of Cape Coast, Cape Coast, Ghana; University of Siena, ITALY

## Abstract

Rapid urbanization and its associated pollution can affect water quality in mosquito breeding habitats and, as a result, the ecology and control of mosquito vectors. To understand the effects of pollution on mosquito vectors, an accurate assessment of water quality in breeding habitats is needed. Presently, water quality assessment of mosquito breeding habitats is usually based on the measurement of individual physicochemical parameters. However, several parameters are sometimes difficult to interpret or may not give a clear picture of the overall water quality of the breeding habitats, especially when the pollutants are in complex mixtures. This study employed the use of Laser-Induced Fluorescence (LIF) spectroscopy to assess water quality in breeding habitats of *Anopheles*, *Aedes*, and *Culex* mosquitoes in urban areas in Cape Coast, Ghana. The LIF spectra, using a 445-nm diode laser, were measured from field-collected water samples in the laboratory. The LIF spectra showed the presence of dissolved organic matter (DOM) and chlorophyll in the breeding habitats. The DOM and chlorophyll fluorescence signals were normalised by the Raman vibrational signals to determine water quality in each habitat. The overall water quality was better in *Aedes* breeding habitats than in *Anopheles* and *Culex* breeding habitats. The poor water quality in *Anopheles* and *Culex* breeding habitats was due to the presence of high fulvic acid and chlorophyll content, which often reflect pollutants from anthropogenic sources. *Anopheles* and *Aedes* habitats were made up of mainly *An*. *coluzzii* and *Ae*. *aegypti* respectively while *Culex* species were identified to genus level. The results add up to the growing concern about the breeding of *Anopheles* in polluted habitats. The study demonstrated for the first time the ability of LIF spectroscopy to assess water quality in mosquito breeding habitats.

## Introduction

Mosquitoes carry and transmit several pathogens to humans making them one of the deadliest insects in the world [1]. About 700 million people are infected by mosquito-borne diseases each year [1]. The major mosquito vectors, *Anopheles*, *Aedes*, and *Culex* species, transmit various pathogens to humans and animals causing several diseases, including malaria, dengue, yellow fever, and various encephalitis. Mosquitoes spend the first part of their lifecycle in stagnant or slow-moving water bodies. *Anopheles* and *Culex* mostly breed in surface waters, including puddles, ponds, and choked gutters, while *Aedes* mosquitoes particularly *Ae*. *aegypti* commonly breeds in containers such as automobile tires and household water storage containers [2]. Notwithstanding, *Culex* and *Anopheles* mosquitoes can also be found cohabiting with *Aedes* mosquitoes in containers [2].

The rapid environmental changes due to urbanisation, climate change, and other anthropogenic activities, especially in developing countries, significantly impact water bodies [3], including mosquito breeding places [4]. These changes have affected the level of water quality in mosquito breeding habitats. Changes in water quality in mosquito breeding sites can affect mosquito vector composition in an area or change their tolerance to insecticides [5–8]. For instance, nutrient pollution and other organic pollutants in aquatic habitats have been found to influence either mosquito diversity or species replacement [6–8]. Such changes can have serious epidemiological consequences on mosquito-borne diseases.

Organic substances constitute the major urban pollutants in freshwater bodies [3] and could come from many sources including domestic activities, traffic, and industries as well as agricultural activities [9, 10]. Common pollutants found in urban water bodies include wastewater and sewage-related discharges, heavy metals, pesticide residues, and organic pollutants including polycyclic aromatic hydrocarbons (PAHs) and polychlorinated biphenyls (PCBs) [9, 10]. In urban areas in Ghana, nutrient pollution resulting from household wastewater and sewage-related discharges is one of the major pollutions in mosquito breeding habitats [11–13]. Surprisingly, *Anopheles* the major malaria vector in the country has been found breeding in such habitats, which were previously known to be inhabited mainly by *Culex* mosquitoes [11, 12]. Similar observations have been reported in other African countries [13, 14]. In order to fully understand the impact of nutrient pollution in breeding habitats on the biology of mosquitoes, there is a need for a reliable technique to assess water quality in mosquito breeding places.

Traditionally, the level of water quality in mosquito breeding habitats is determined by measuring several physical and chemical characteristics of water from the mosquito breeding habitats [11–16]. These measurements are taken in situ and/or through laboratory analysis of water collected from the breeding habitats. In the laboratory, these analyses can be time-consuming, laborious, and expensive. Also, the determination of water quality through the measuring of several parameters sometimes becomes difficult to interpret or may not give a clear picture of the water quality status of the breeding habitats [15]. For example, in a water quality assessment study in mosquito breeding habitats in Yaoundé (Cameroun), the two groups of habitats that were described as polluted and non-polluted only differed in conductivity; the rest of the physicochemical parameters were similar including ammonia and dissolved oxygen, which are usually used as indicators of organic pollution [15]. Their study and other studies from different countries [11–14, 16] highlight the difficulty in interpreting several physicochemical parameters to determine water quality status in mosquito breeding habitats. An alternative technique that is reliable and easy to interpret would help improve water quality assessment in mosquito breeding habitats.

In this study, we explored the use of a Laser-Induced Fluorescence (LIF) spectroscopy to determine nutrient pollution in mosquito breeding habitats in Cape Coast, Ghana. The degree of nutrient pollution or eutrophication can be measured by the level of chlorophyll in the water [17, 18]. Besides chlorophyll, the content of dissolved organic matter (DOM) in water bodies is another important water quality indicator [19, 20]. DOM is the largest component of organic matter in the aquatic system and its levels and composition can be greatly influenced by nutrient pollution [21]. Both chlorophyll and DOM were measured together in this study to evaluate water quality in mosquito breeding habitats [22]. From a LIF data of water samples from a mosquito breeding habitat, the following distinct spectral signals, i.e. water Raman signal, the DOM signal, and the chlorophyll signal were isolated and used to determine the level of water quality in the mosquito breeding habitat using a procedure described by Lu and his colleagues [22].

## Materials and methods

### Study sites

This study was conducted in Cape Coast, a coastal city in Ghana, about 165 km west of Accra, the national capital. The town is within the coastal savannah ecological zone, with the major rainy season between May and July and the mean monthly relative humidity varying between 85 and 99%.

### Larval survey

**Fig 1:**
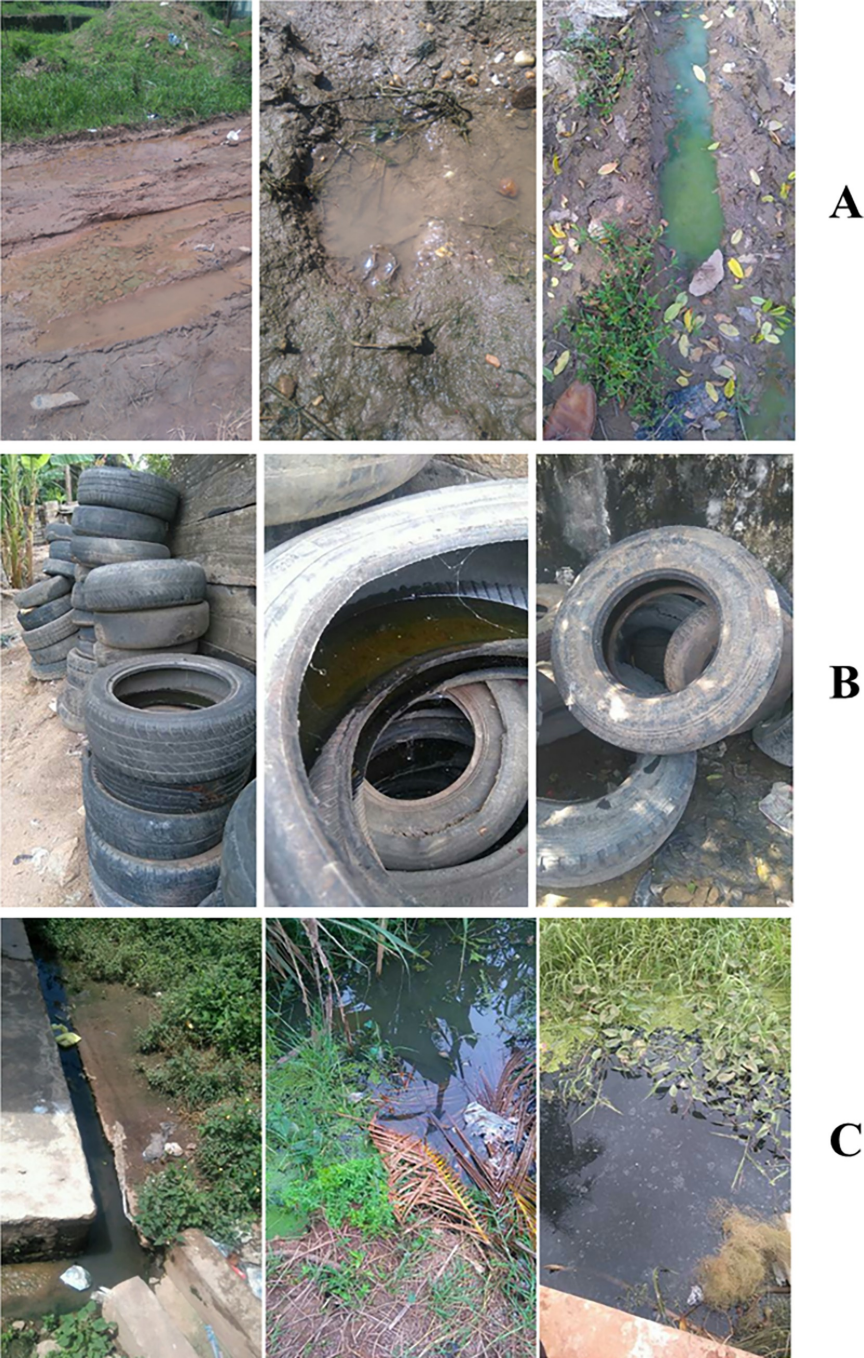
Breeding habitats of A. *Anopheles* (*An*. *coluzzii)*, B. *Aedes* (*Ae*. *aegypti)*, and C. *Culex* mosquitoes where water was collected and measured for Laser-Induced Fluorescence spectra.

A larval survey was conducted in Cape Coast Metropolis between 2015 and 2017 to identify typical breeding habitats of *Anopheles*, *Culex*, and *Aedes* mosquitoes. Larvae and water samples were collected from the habitats and sent to the laboratory. The mosquito larvae were fed on a standard fish meal (Tetrafin®) and reared to adults. The adults were used for species identification. Water samples were collected in 8 ml glass vials and transported to the laboratory in a dark and airtight container with ice packs to prevent bacteria growth and photo-degradation. *Anopheles* breeding habitats were mainly water puddles or small, temporary, and surface water bodies. *Culex* breeding habitats were also mainly choked gutters and cesspits containing wastewater from houses or industries, while *Aedes* breeding habitats were all abandoned automobile tires (Fig 1). Water samples were taken from forty-three breeding sites, which consisted of twenty-four *Anopheles* breeding sites, eight *Aedes* breeding sites, and eleven *Culex* breeding sites. The larvae that were reared to adults were morphologically identified; *Culex* mosquitoes were identified to the genus level while *Aedes* and *Anopheles* were identified to the species level with the aid of PCR [11, 23]. Results from the PCR, which has been reported in another study [11, 23] showed that all the *Anopheles* and *Aedes* species were *An*. *coluzzii* and *Ae*. *aegypti*, respectively.

### Laser-Induced Fluorescence (LIF) spectroscopy measurement

**Fig 2:**
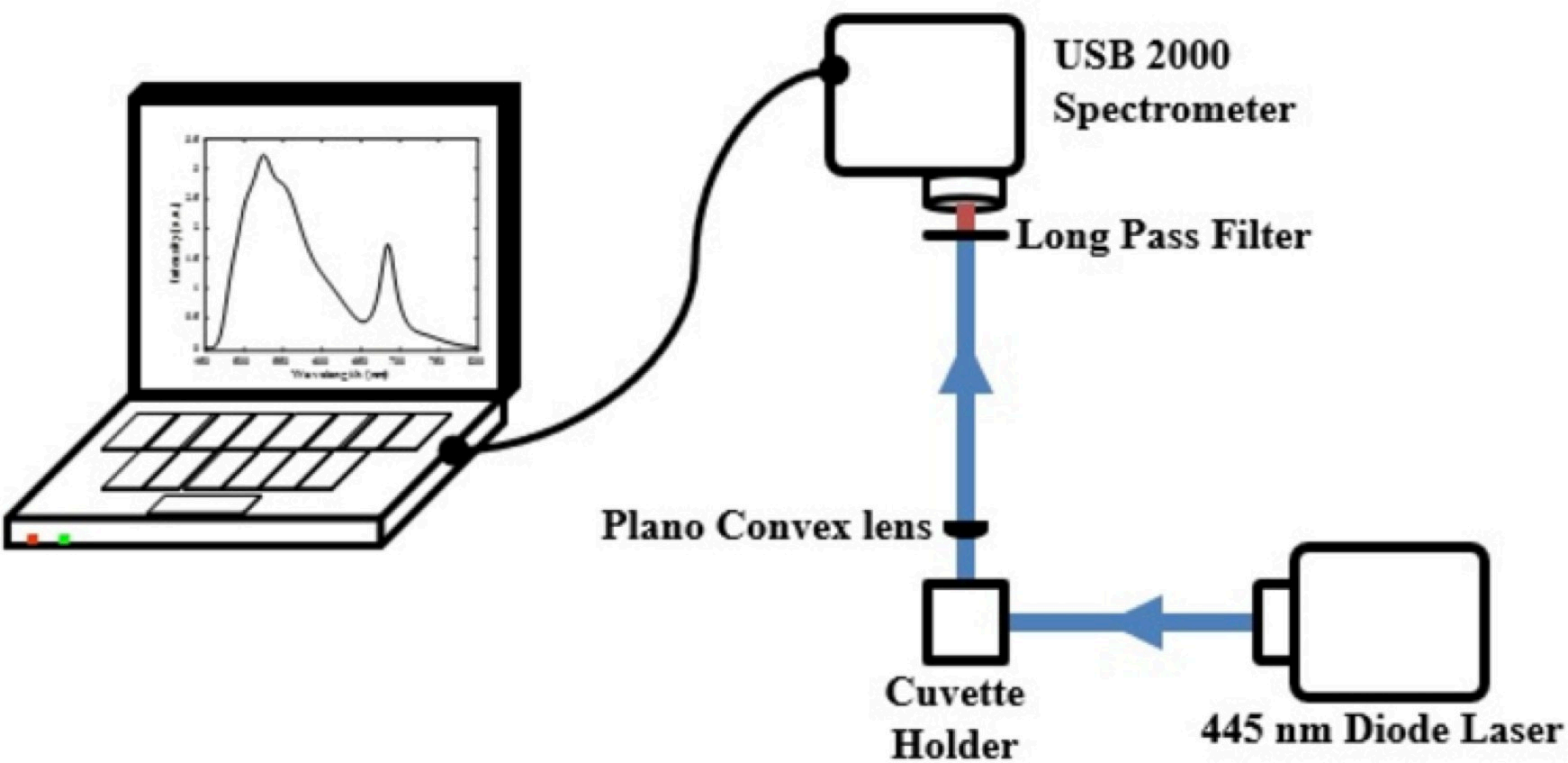
Experimental setup used to measure the LIF spectra of water samples from the mosquito breeding sites.

At the laboratory, water samples were measured for their LIF spectra using the experimental setup shown in Fig 2. A 100-mW diode laser (O-like, China) operating at 445 nm was used as excitation for inducing the fluorescence, and a compact spectrometer (USB 2000, Ocean Optics) was used as the detector. The spectrometer was first calibrated using a 632.8 nm HeNe laser and confirmed with the 445 nm laser. The excitation source, detector, and samples were arranged in a 90^o^ setup configuration. After its interaction with the sample, the emitted light was collected with a plano-convex lens and focused on the spectrometer. A long-pass absorptive edge filter (GG-445, Edmunds) was used to cut out and allow only photons with wavelengths higher than 450 nm to be transmitted into the spectrometer. The spectrometer collected an average of 90 spectra for each sample within 60 sec for an integration time of 300 msec over the spectral range of 200 to 900 nm.

### Data analysis

The measured LIF spectra of each water sample were first preprocessed according to Eq (1) by the standard normal variate (SNV) method [24]. This method of normalisation has the purpose of making all spectra comparable in terms of intensities. SNV provides an efficient way of eliminating constant baseline effects and scaling differences from the spectra for comparison. It normalises the spectral data by subtracting each spectrum (*F_ij_*) by its mean (${\bar{F}}_{j}$) and dividing by its standard deviation (*S_j_*)

$${\overset{˜}{F}}_{ij}=\frac{F_{ij}-{\bar{F}}_{j}}{S_{j}}$$ (1)

After pre-processing with SNV, all the spectral data from the various mosquito breeding sites were saved as a single file. This was used as input data for the different analyses employed in this study. The ratios of the area covered by fluorescence emission from DOM, OH molecules of water Raman vibrational band, and Chlorophyll bands are plotted for comparison among the different species of mosquitoes as described by Lu et al. [22].

Spline interpolation was used in fitting each of the LIF spectra to separate the major components for this analysis. Spline interpolation of spectral data involves the estimation of values to generate a curve using a piecewise function of cubic polynomial segments connected in a given interval. Weights of the coefficients on a series of unique cubic polynomials are fitted between each of the data points to interpolate the data. The coefficients ensure the curve passes through each of the data points without any breaks in continuity. The first and second derivatives of this segment match those of the adjacent segments at the shared endpoint.

The PCA method was applied to the input SNV normalised data to transform each sample’s spectrum as a single point in PC space [25]. This is a way of reducing the number of potential dimensions in the LIF spectra that need to be studied for characterising the breeding sites of the different species of mosquito. PCA first finds the measure of how each input data ${\overset{˜}{F}}_{ij}$ is associated with one another calculated as the Covariance matrix $G_{jj}^{T}$ as shown in Eq (2) and then finds the directions in which the input data ${\overset{˜}{F}}_{ij}$ are dispersed according to Eq (3).


$$G_{jj}^{T}={\overset{˜}{F}}_{ij}{\overset{˜}{F}}_{ij}^{T}$$ (2)

$$E_{jj}^{T}={\overset{˜}{F}}_{ij}G_{jj}^{T}$$ (3)

From Eq (3) $G_{jj}^{T}$ is the eigenvector matrix and the columns of the matrix $E_{jj}^{T}$ denotes the eigenvalues known as the principal components which provide the relative importance of these different directions. The eigenvalues are used for the score and the eigenvector is used for the loadings plot. The PCA scores are based on the variance across the data set as a whole. The scores are used to detect underlying patterns or groupings of samples in a large data set with the associated loadings giving information on the spectral wavelengths leading to the patterns.

PeakFit software (SYSTAT Software Inc., version 4.12) [26], was used to deconvolute and fit the overlapping spectral bands defined by the PCA loadings. A Gaussian function was selected for the fitting of the spectral curves. The second derivative method and a 5.55% Savisty-Golay filter were selected in the software and applied to the spectra before the spectral deconvolution. The software uses a least square minimization iteration to ensure that the r^2^ (coefficient of determination) value of the fitted spectra always reached larger than 0.995 before the fitting routine is ended.

## Results

**Fig 3:**
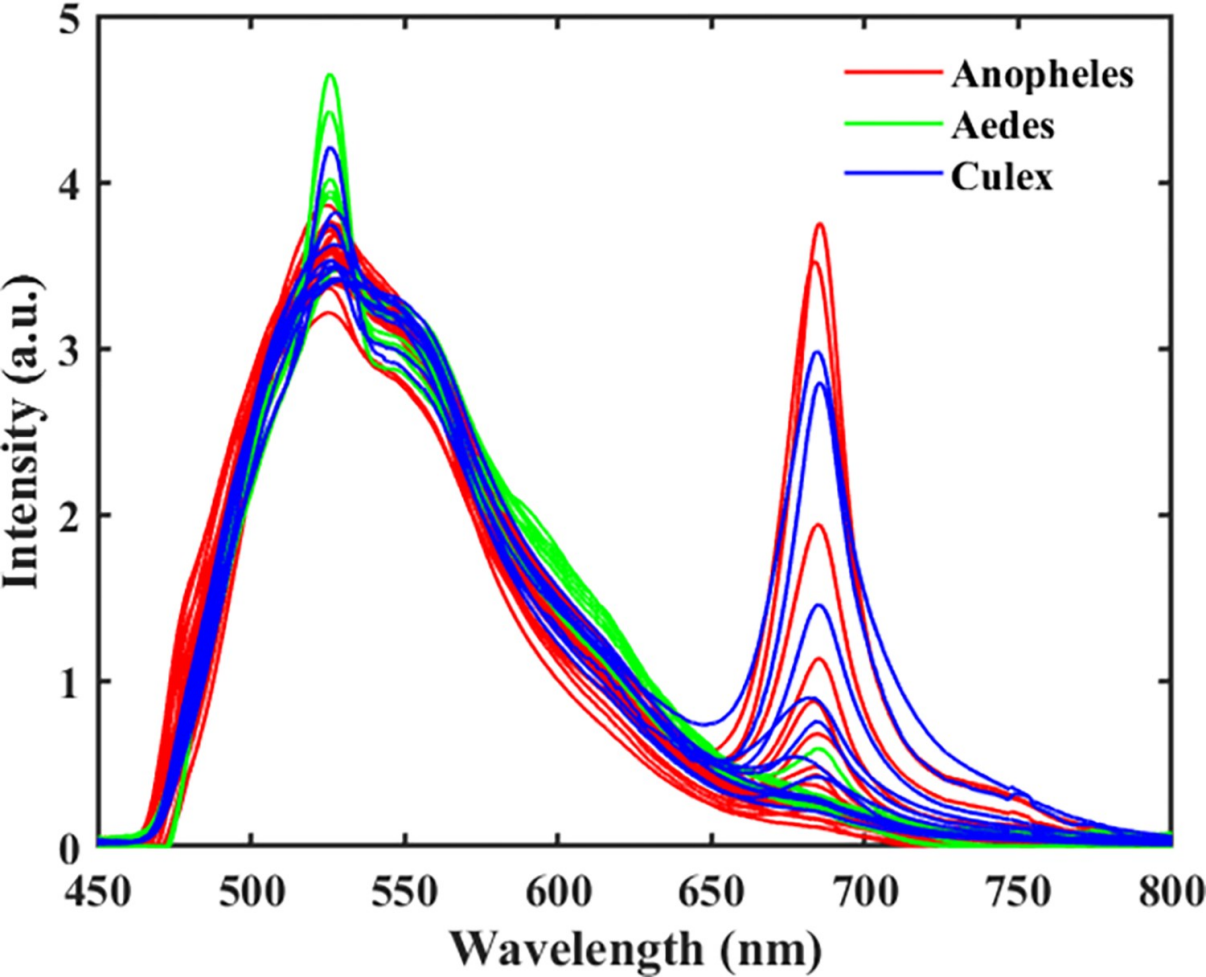
LIF spectra of water samples collected from three different breeding sites of mosquito species (*Anopheles*, *Aedes*, and *Culex*) in Cape Coast, Ghana.

**Fig 4:**
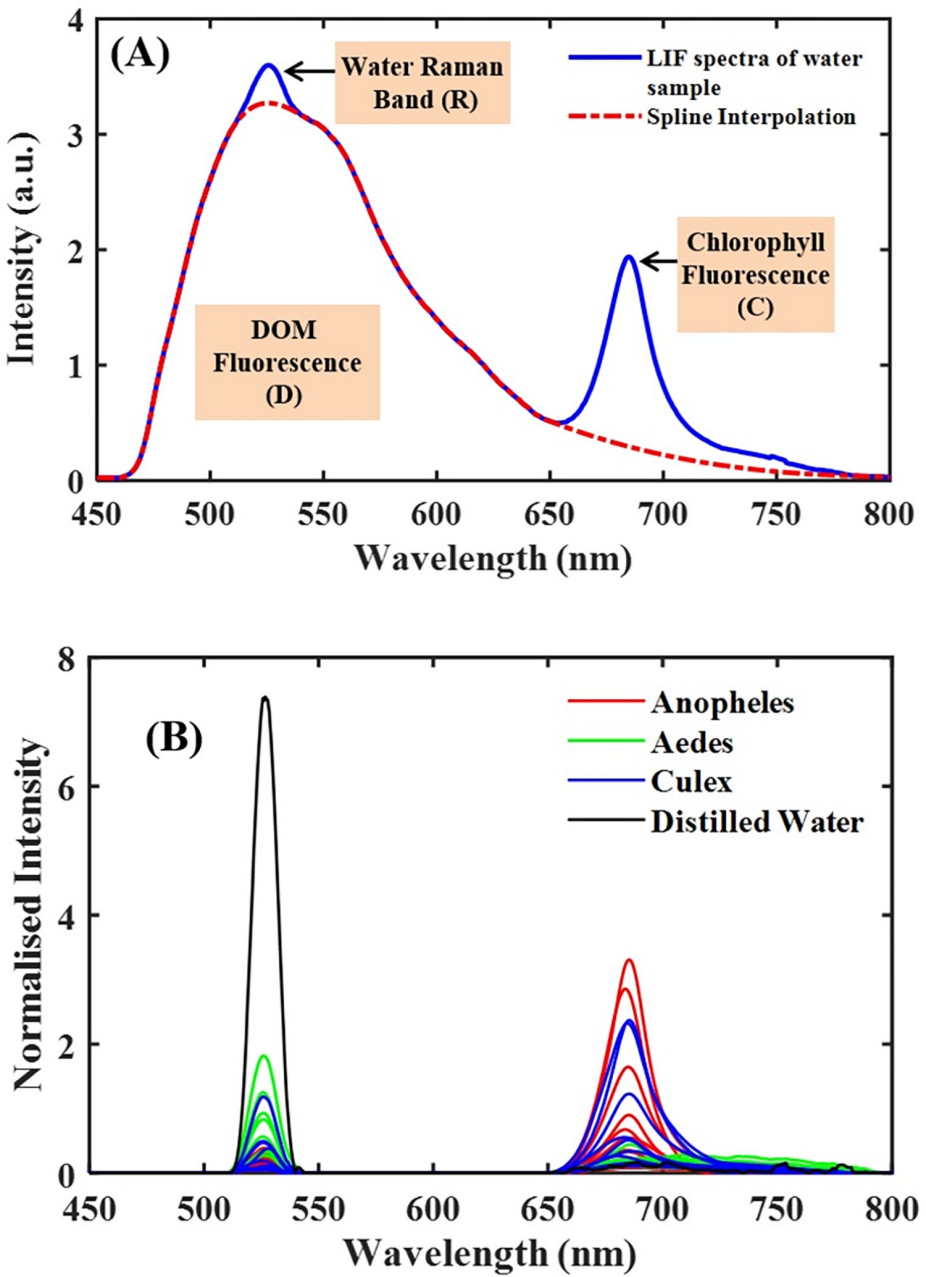
A-Fitted LIF spectra showing the DOM, water Raman band and chlorophyll fluorescence after spline interpolation separately. B- the water Raman and chlorophyll band for the various mosquito breeding sites after subtracting the DOM.

**Fig 5:**
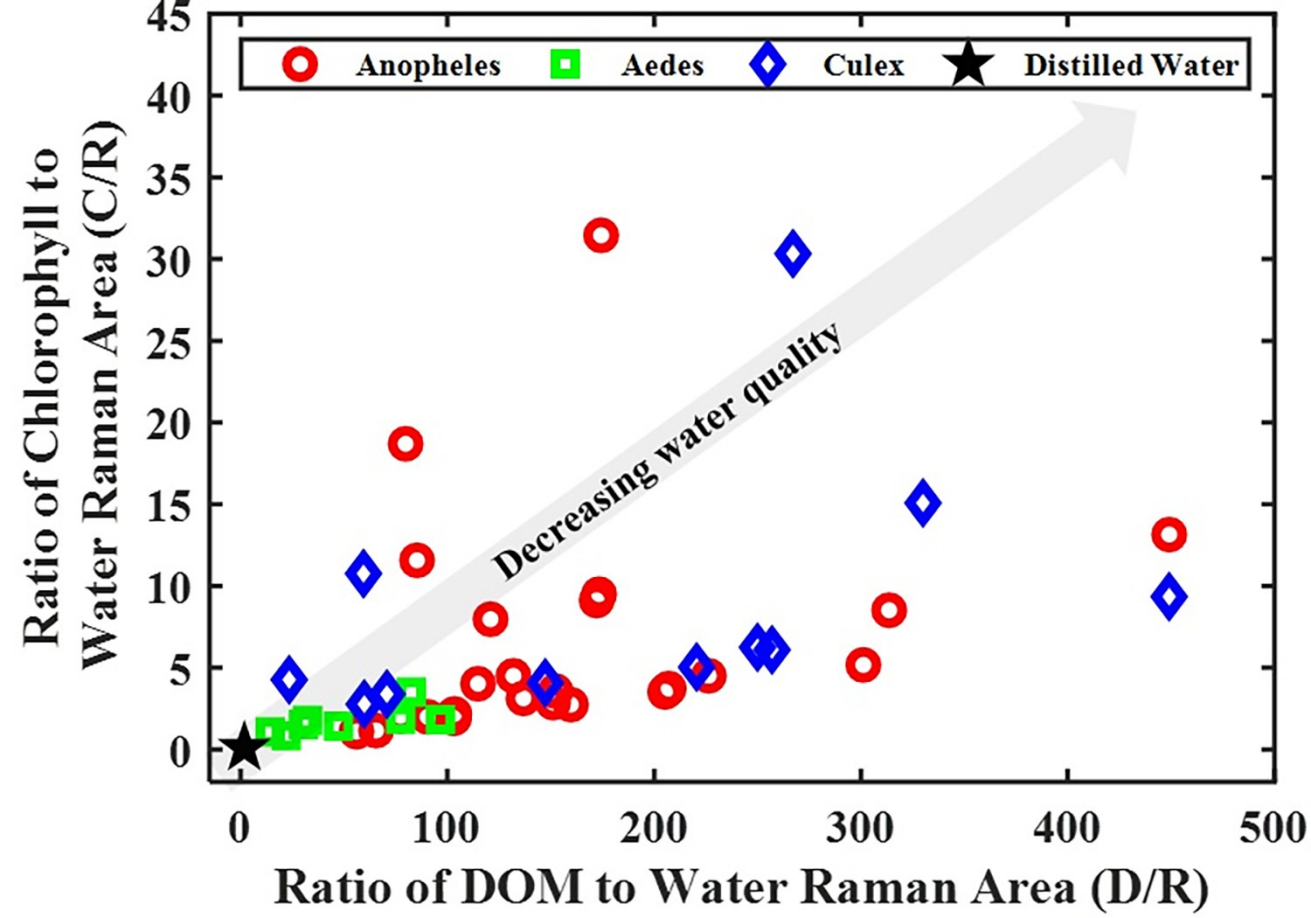
The relationship between the Chlorophyll to water Raman band ratio (C/R) and DOM to water Raman band ratio (D/R) for water samples from the mosquitoes’ various breeding habitats. It enables the determination of the water quality of the samples from multiple breeding sites.

The LIF spectra result comprised two prominent emission bands, 450–650 nm, and 650–800 nm, emanating mainly from dissolved organic matter (DOM) and chlorophyll fluorescence, respectively (Fig 3). Atop the DOM region is a moderately narrow peak centered around 523 nm, representing the -OH molecules’ Raman vibrational band. For clean water (i.e., distilled water), this narrow peak is singly observed in the LIF spectrum (Fig 4). The water quality status of the breeding habitats determined by the ratio of DOM to Raman (D/R) band and Chlorophyll to Raman Band (C/R) are shown in Figs 4 and 5. The clean water (indicated with a black star) is at the origin (0,0) of the graph, where the values of D/R and C/R are negligible. The quality of water thus decreases with increasing distance from the origin of the graph. From Fig 5, it can be observed that water samples from *Aedes* breeding habitats were clustered and closer to distilled water (the origin), while water samples from *Anopheles* and *Culex* habitats were scattered, showing different water quality levels. From the results, the deteriorating water quality was due to high chlorophyll levels in some of the breeding habitats.

**Fig 6:**
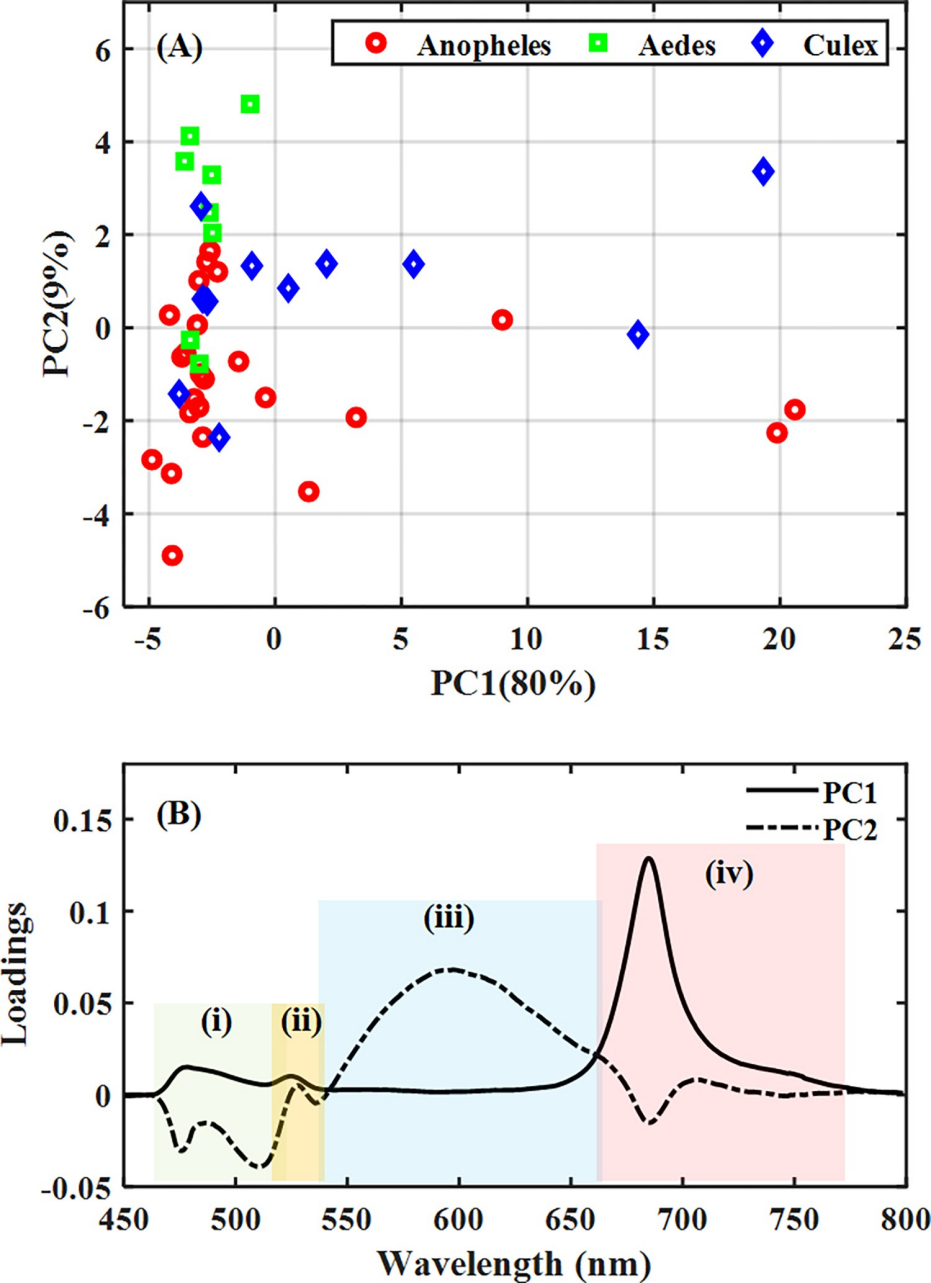
PCA of LIF spectra of water samples from the various breeding sites with (A) Score plot showing distribution pattern among samples and (B) loading plot for PCs 1 and 2 showing specific regions (wavelengths) causing the distribution pattern in the score plot; (i) 460 nm– 515 nm, and (iii) 535 nm– 660 nm representing the DOM region, (ii) 515 nm– 535 nm representing the water Raman band and (iv) 660 nm– 780 nm representing the Chlorophyll region.

**Fig 7:**
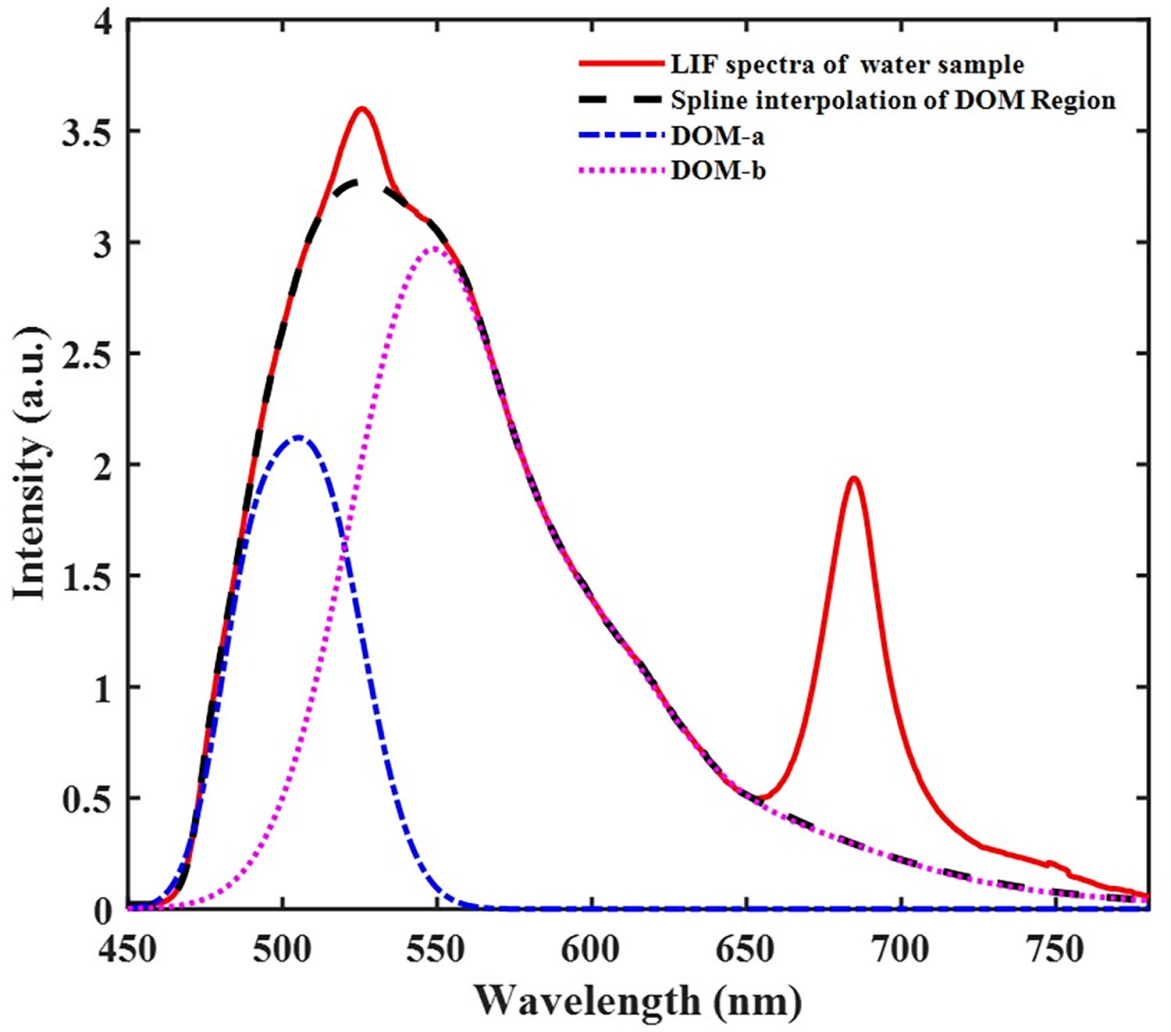
Deconvolved LIF spectra showing the constituents of water samples from mosquito breeding sites.

The combined PC1 and PC2 loadings show compartmentalization of the broad fluorescence spectrum into four sections; (i) 460–515 nm, and (iii) 535–660 nm representing the DOM region, (ii) 515–535 nm representing the water Raman band and (iv) 660–780 nm, representing the Chlorophyll region (Fig 6A and 6B). The combined PC1 and PC2 accounted for 89% variations in the data set. The loadings showed that wavelength variables from the chlorophyll region (iv) were the essential component causing the distribution observed along the PC1 axis. The DOM region, which was made up of two peaks (i) DOM-a and (iii) DOM-b, on the other hand, was also responsible for the order of distribution along the PC2 axis. Along the PC2 axis, the DOM-b region had positive loadings, whereas the DOM-a region recorded negative loadings. By relating the loadings to the score plot, it was observed that water from most of the breeding habitats of *Aedes* occurred at the positive side of PC2, and water from most of the *Culex* and *Anopheles* habitats was found scattered. Interestingly, both *Anopheles* and *Culex* found on the positive side of the PC2 axis had a lower score than the *Aedes* mosquitoes. The fluorescence spectra were replotted with the DOM fractions deconvoluted to depict DOM-a and DOM-b, as shown in Fig 7. The DOM-a and DOM-b are related to fulvic acid and humic acid fractions, respectively. This observation is so because of the difference in molecular weights based on the emission wavelengths [27, 28]. Interestingly, all the *Aedes* breeding habitats were mainly dominated by humic acid compounds, whereas the breeding habitats of *Anopheles* and *Culex* mosquitoes had different components of the DOM fractions.

## Discussion

Mosquito breeding habitats in urban areas are under the significant influence of various anthropogenic pressures, including organic and nutrient pollution [3, 4, 7]. This condition can facilitate the replacement of native mosquito species in the urban areas or influence the adaptation of species to the polluted breeding habitats. A typical example is the recent adaptation of *Anopheles* (*An*. *gambiae* s.l.) into breeding in nutrient-rich habitats, which were previously known to be inhabited mainly by *Culex* mosquitoes [11–16]. This phenomenon could compromise vector control strategies. To be able to fully understand the impact of such adaptations on the biological fitness of mosquito vectors, an accurate determination and characterization of nutrient pollution of individual habitats are needed. As earlier stated, the traditional method of assessing water quality in mosquito breeding habitats has inherent limitations [11–16]. On one hand, single parameters do not give a clear picture of the level of water quality with regards to nutrient pollution of a breeding habitat. On another hand, when several parameters are measured, the interpretation of the level of water quality becomes difficult [15]. The difficulty becomes even more pronounced when the level of water quality has to be compared among individual habitats [15]. Addressing this problem would require a technique that cannot only give a better overview of the level of nutrient pollution but also a better interpretation of the level of water quality in the breeding habitats. From the results, the LIF spectra detected the presence of dissolved organic matter (DOM) and chlorophyll in the breeding habitats. This result suggests the presence of nutrient pollution in the breeding habitats since chlorophyll content is an important indicator of nutrient pollution. When the DOM and chlorophyll fluorescence signals were normalized by the Raman vibrational signals, the general water quality was found to be better in *Aedes* breeding habitats than in *Anopheles* and *Culex* breeding habitats. The Raman vibrational signal served as a built-in reference to correct any interference from changes in optical attenuation [29]. Clean water has zero-normalized DOM and chlorophyll signals and thus, increasing ratios of some of the breeding habitats indicated deteriorating water quality or increasing levels of nutrient pollution. DOM and chlorophyll measured in this study represent a broad spectrum of nutrient and other organic compounds and thus, gives a more realistic level of nutrient pollution than individual physicochemical parameters that are often used. Furthermore, the level of pollution was clearly defined and more comparable among individual habitats than the interpretation of results from physicochemical parameters.

DOM may originate from a wide range of natural and anthropogenic activities [30, 31]. The fractions of DOM from aquatic sources are broadly classified as protein and humic substances. Humic compounds are also subdivided into fulvic acid and humic acid [32]. Interestingly, all the *Aedes* breeding habitats were mainly dominated by humic acid compounds, whereas the breeding habitats of *Anopheles* and *Culex* mosquitoes had different components of the DOM fractions. Humic fractions occur naturally from plant materials’ breakdown, while fulvic acid is associated with natural and anthropogenic sources, including crude sewage [19]. In this study, the *Culex* breeding habitats were mainly choked gutters and cesspits containing liquid waste from households and industries, whiles *Anopheles* habitats were also mainly of water puddles that receive run-off from stormwater and other drainage systems. It is therefore not surprising that high levels of fulvic acid and chlorophyll were found in both *Anopheles* and *Culex* habitats. This observation is consistent with a previous study in Cape Coast, where *Anopheles* mosquitoes were found cohabiting with *Culex* species in polluted organic habitats, which was indicated by low dissolved oxygen and high ammonia levels [11]. The result is also consistent with other studies in urban areas that have found *Anopheles* in nutrient-rich breeding habitats [11, 13, 14]. Notwithstanding, the levels of water quality in both *Anopheles* and *Culex* breeding habitats varied.

This study presents the first attempt to use LIF to study water quality in mosquito breeding habitats. The study’s main focus was on nutrient pollution. However, a laser with an appropriate excitation wavelength can be used to detect other several pollutants including. PAH and heavy metals. Indeed, PAH compounds such as pyrene and phenanthrene have emission in the range of 450–550 nm when excited with wavelength up to 450 nm [33]. However, the fluorescence intensity of these compounds would not be that high in the current study, given the excitation wavelength used (445 nm). Further studies with LIF on other pollutants in mosquito breeding habitats are therefore recommended. Accurate characterisation of water pollutants in mosquito breeding habitats is a prerequisite for any field studies to elucidate the impact of breeding in polluted water on the biology of mosquito vectors. Already, different field studies have shown some beneficial impacts of DOM on mosquito life traits [34, 35]. For instance, high DOM concentration has been hypothesised to enhance food resources through stimulation of the microbial food web [34]. Berry et al. [35] also found that DOM protects mosquito larvae by shading them from damaging solar UV radiation. On the other hand, the breeding of *Anopheles* in organically polluted waters particularly with heavy metals has been shown to either have an adverse effect on ecological fitness [36] or enhance vector tolerance to insecticides [37]. *Anopheles* mosquitoes were made up of mainly *An*. *coluzzii* in this study. The results support the growing concern about the breeding of *An*. *coluzzii* in polluted habitats. Until recently, *An*. *coluzzii* and *An*. *gambiae* existed as two molecular forms within *An*. *gambiae* senso stricto. The evolution (speciation) of the two molecular forms into separate species had been linked to the adaptation of *An*. *coluzzii* into anthropogenic xenobiotics and the expansion of their niche to polluted habitats whereas *An*. *gambiae* appears to have maintained in rain-dependent temporary breeding sites [38, 39]. Thus, the study of water quality or pollution in *Anopheles* breeding habitats are of paramount importance in gaining a deeper understanding of the biology and control of these vectors.

## Conclusion

We have demonstrated for the first time LIF spectroscopy as a suitable technique to study water quality in mosquito breeding habitats. The technique was able to determine and characterise pollution simultaneously in the breeding sites. The level of water quality was clearly defined, which makes it easy to compare among the habitats. The DOM and chlorophyll measured in this study represent a broad spectrum of organic compounds. In urban areas where pollutants are usually in complex mixtures, the use of broad spectrum parameters may give a more realistic water quality status than individual physicochemical parameters that are often used. Employing this technique together with the existing methods could help gain better understanding on the role of water quality in the development of mosquito larvae. Also, the technique has the advantages of high speed and sensitivity and can be incorporated in a hand-held device to measure water quality *in situ* or applied in remote sensing of breeding habitats.

## Supporting information

S1 Appendix Dataset for laser-induced fluorescence spectra of water from mosquito breeding habitats in Cape Coast. (XLS)
